# Structure-based knowledge acquisition from electronic lab notebooks for research data provenance documentation

**DOI:** 10.1186/s13326-021-00257-x

**Published:** 2022-01-31

**Authors:** Max Schröder, Susanne Staehlke, Paul Groth, J. Barbara Nebe, Sascha Spors, Frank Krüger

**Affiliations:** 1 Institute of Communications Engineering, University of Rostock, Rostock, Germany; 2 University Library, University of Rostock, Rostock, Germany; 3 Department of Cell Biology, University Medical Center Rostock, Rostock, Germany; 4 Informatics Institute, University of Amsterdam, Amsterdam, Netherlands; 5 Department Life, Light & Matter, University of Rostock, Rostock, Germany; 6 Department Knowledge, Culture & Transformation, University of Rostock, Rostock, Germany

**Keywords:** Research data, Provenance, Knowledge acquisition, Electronic laboratory notebooks, Semantic documentation, RO-Crate, FAIR

## Abstract

**Background:**

Electronic Laboratory Notebooks (ELNs) are used to document experiments and investigations in the wet-lab. Protocols in ELNs contain a detailed description of the conducted steps including the necessary information to understand the procedure and the raised research data as well as to reproduce the research investigation. The purpose of this study is to investigate whether such ELN protocols can be used to create semantic documentation of the provenance of research data by the use of ontologies and linked data methodologies.

**Methods:**

Based on an ELN protocol of a biomedical wet-lab experiment, a retrospective provenance model of the raised research data describing the details of the experiment in a machine-interpretable way is manually engineered. Furthermore, an automated approach for knowledge acquisition from ELN protocols is derived from these results. This structure-based approach exploits the structure in the experiment’s description such as headings, tables, and links, to translate the ELN protocol into a semantic knowledge representation. To satisfy the Findable, Accessible, Interoperable, and Reuseable (FAIR) guiding principles, a ready-to-publish bundle is created that contains the research data together with their semantic documentation.

**Results:**

While the manual modelling efforts serve as proof of concept by employing one protocol, the automated structure-based approach demonstrates the potential generalisation with seven ELN protocols. For each of those protocols, a ready-to-publish bundle is created and, by employing the SPARQL query language, it is illustrated that questions about the processes and the obtained research data can be answered.

**Conclusions:**

The semantic documentation of research data obtained from the ELN protocols allows for the representation of the retrospective provenance of research data in a machine-interpretable way. Research Object Crate (RO-Crate) bundles including these models enable researchers to easily share the research data including the corresponding documentation, but also to search and relate the experiment to each other.

## Background

Effective reuse of research data requires comprehensive documentation of their provenance. Beside metadata, knowledge about the generating process helps to understand research data and allows for the reproduction of research investigations. This includes sources of input data such as parameters and assumptions but also information about instrumentation, devices and materials. For wet-lab experiments such knowledge is increasingly documented in ELNs. The focus of these tools is the documentation of laboratory activities that produce research data in so-called ELN protocols. In addition to this textual description, the FAIR principles [1] provide general guidance on research data documentation in terms of metadata. However, they do not prescribe technical details about the implementation of such documentation [2].

To foster the realization of the FAIR principles for research data produced in wet-lab experiments, we aim for machine-interpretable representations of experimental documentation of the process that is the origin of the data. In other words, the provenance information about the research data including the activities and involved researchers, resources and equipment should be semantically represented. For this purpose, we employ the frequently used [3] PROV W3C recommendation [4], which ontologically, in PROV Ontology (PROV-O), defines entities, activities, and agents including their relations. In particular, according to Belhajjame et al. an entity is a “*physical, digital, conceptual, or other kind of thing with some fixed aspects*” [5], an activity is “*something that occurs over a period of time and acts upon or with entities; it may include consuming, processing, transforming, modifying, relocating, using, or generating entities*” [5] and an agent is “*something that bears some form of responsibility for an activity taking place, for the existence of an entity, or for another agent’s activity*.” [5] With respect to wet-lab experiments, all biological and chemical resources as well as the devices and software but also the research data itself can be seen as entities; researchers conducting the experiment are the agents and the process of the research data creation consists of activities. The semantic representation of this information as a Knowledge Graph (KG) [6] can be achieved by the use of modern web-technologies where the terms and their relations are defined in ontologies such as PROV-O (TBox modelling), the instances are building up the KG (ABox modelling) and other KGs can be linked in order to create an interconnected graph of semantic knowledge.

In this paper, we aim at an automatic extraction of information from ELN protocols in order to transfer them into a semantic representation that documents the produced research data. For this purpose, we employ the documentation of Calcium imaging (Ca-imaging) experiments originally proposed by Staehlke et al. [7] as a running example. In particular, we use ELN protocols that document the conduction of Ca-imaging experiments in order to: (i) demonstrate the feasibility of manually transferring an ELN protocol into a semantic representation encoding the provenance of research data, (ii) automate the information extraction and modelling by exploiting the structure of an ELN protocol by means of a structure-based approach, and (iii) evaluate the proposed method by answering provenance questions from the resulting bundle of research data and the corresponding semantic model.

**Fig. 1:**
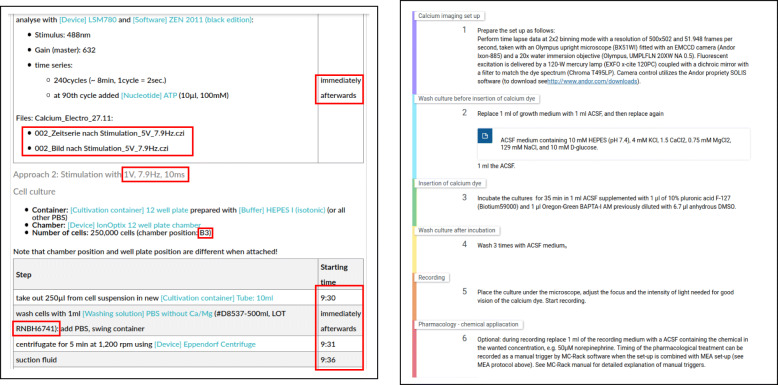
Excerpts of an ELN protocol that represents a particular experiment including all details such as timestamps, lot numbers as well as the research data (left) and a protocol template containing general instructions of experiments without these details (right, source [9])

Here, the term ELN protocol refers to the actual documentation of the wet-lab experiment within an ELN and is different to the term protocol templates that encode instructions to be performed in order to conduct particular procedures, as for instance published at https://www.protocols.io/. While those protocol templates do encode a list of abstract instructions, they do not necessarily reflect particular research data, nor instrumentation, parameters or other to the execution specific information. ELN protocols, in contrast, represent the documentation of the actual experiment and the contained information is thus necessary to understand how the resulting research data was generated. This includes manufacturer specific information about resources used in the experiment such as lot^1^ numbers. Furthermore, passage numbers of the resources, the times when an activity was conducted, the parameters used in a device as well as the research data and the researchers conducting the experiment are information specific to a particular experiment. Figure 1 illustrates the differences by providing an example for an ELN protocol and a protocol template.

The work presented here is based on a preliminary investigation regarding the effectiveness of manually modeling ELN protocols by use of ontologies [8]. Here, we extend this preliminary work by discussing the potential of *automatic information extraction* from ELN protocols by employing structural information and discussing the differences and implications of both approaches. Moreover, while the previous work only sketched the semantic representation of the wet-lab experiments, here, we focus on the generation of ready-to-publish research data bundles including the semantic description of the origin of the research data.

## Use case

To demonstrate the feasibility of the proposed approach, a typical wet-lab investigation was chosen as a use case. In the following, we introduce the use case and derive questions regarding the provenance of the corresponding research data.

### Biomedical wet-Lab experiments

The objective of the biomedical study was to investigate the intracellular calcium ions (Ca^2+^) dynamics by Calcium-imaging (Ca-imaging) under different settings [7]. In particular, two different wet-lab experiments were considered: (i) an investigation of the influence of different material surface conditions on Ca^2+^ mobilisation, and (ii) an investigation regarding the Ca^2+^ dynamics under the influence of electrical stimulation. Both types of experiments involve similar activities of the researchers. In particular, each experiment employs the Ca-imaging method previously established by Staehlke et al. [7] in different settings. The particular conditions, e. g., surface conditions or parameters of the electrical stimulation, are investigated within each experiment, while the order of the different variations was permuted across the experiments. That is, after a preparation phase, where all materials and devices are prepared, the same procedure, i. e., Ca-imaging, was executed for the different conditions. During the experiment several materials and devices are employed, such as cell line passages, buffer, and microscopes.

**Fig. 2:**
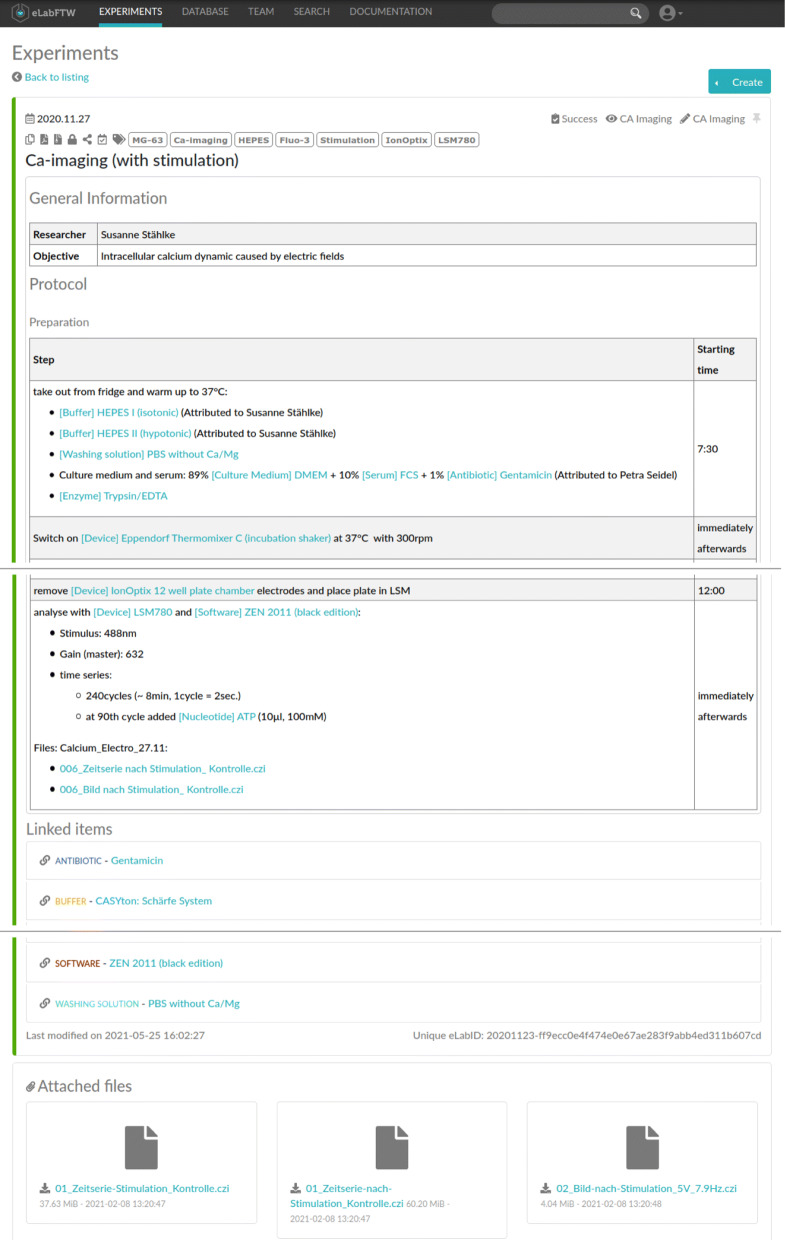
ELN protocol about a Ca-imaging experiment in the elabFTW software. It contains general information (top), the list of activities with their starting time (middle), used inventory items, and uploaded research data (bottom)

For the purpose of this study, we asked the researchers to use an ELN for the documentation of their wet-lab activities, resulting in eight ELN protocols, one for the first experiment and seven for the latter, representing different permutations of the sequential execution of Ca-imaging for different electrical stimulation parameters. In particular, elabFTW (Deltablot, https://www.elabftw.net/, v3.6.7) [10] a domain-independent ELN was used. Figure 2 shows an excerpt of a protocol from the use case.

**Fig. 3:**
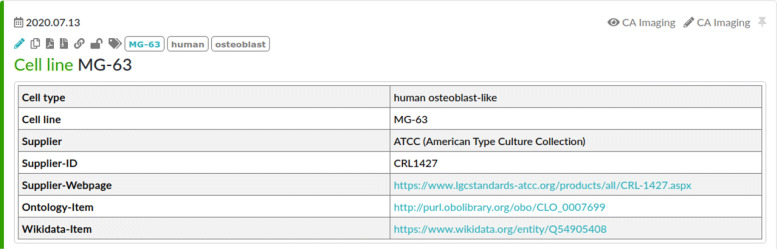
Shortened documentation of a Ca-imaging experiment in the elabFTW ELN software. The upper part contains general information about the investigation, followed by the list of activities with their starting time. Below, used inventory items and uploaded research data are listed

ELNs often provide an inventory database that allows the maintenance of materials and other research resources used during the experiments. Typically, each resource belongs to a configurable set of categories, e. g., cell lines, buffer, software, or devices. These entries in the inventory database can be linked from within the protocol when used within the corresponding experiment. Figure 3 illustrates the entry to the inventory database for the MG-63 cell line that is used in the experiments of the use case. Note that this entry is already augmented by information about ontology classes that were added during the manual model engineering process. Here, we use such ontology references but also other resource identifiers, such as Research Resource Identifiers (RRID) (https://scicrunch.org/resources), could be used for resource reference. However, these RRIDs do not reflect different versions of the resources, e. g., when describing a software. Thus, they can be used to annotate the inventory database of the ELN similar to the ontology classes, but cannot be used on their own. Research data is attached to the ELN protocol by uploading and linking from within the textual description of the step that describes the generating activity.

In summary, the execution of an individual experiment took about 4.5 hours resulting from the preparation and the sequential executions of the Ca-imaging procedure under five different stimulation settings consisting of 15 steps, each. Each protocol referred to 22 inventory items in the database and between 85 and 110 data files of different types were generated. The different file types include (i) CZI files (developed by ZEISS) containing the microscope settings and the recorded images and raw measurement data, (ii) image files in JPEG format to illustrate particular excerpts from the video recordings, and (iii) raw measurements of the luminescence over time in the form of XML encoded tabular data files. The latter two formats are exports from the CZI files. The provenance of all attached files needs to be documented.

### Research data provenance

When considering this use case, several questions regarding the provenance of the research data can be raised. To this end, we consider questions based on the W7 provenance model [11], that describes provenance as combinations of *What*, *When*, *Where*, *How*, *Who*, *Which* and *Why*. We consider each question individually, encoding the view of a researcher that aims at re-using the research data from our use case. The questions were developed together with the domain experts and resemble actual questions that arise when considering the replication of the documented experiments.

1. *Who* participated in the study?
  *With respect to the provenance of research data, all researchers contributing to the creation are of interest, i. e., we expect to get a list of all researchers and their affiliations involved in an experiment.*
2. *Which* biological and chemical resources and which equipment was used in the study?
  *In particular, we are interested in the resources and the equipment used in an experiment including all details such as the lot number and the passage information.*
3. *How* was a particular file created?
  *What was the sequence of activities that led to the creation of a particular file is a question that might help other researchers in comprehending the data.*
4. *When* was an activity conducted?
  *The date and the time point of a particular activity but also its duration are of interest. This information is useful for the planning of similar experiments, but also with respect to the comprehensibility of the results as the date and time point might influence them, e. g., due to weather or other environmental phenomena.*
5. *Why* was the experiment done?
  *Understanding why the research data was created is crucial for their comprehensibility. We take the objective of the experiment as the reason for the creation.*
6. *Where* was the experiment conducted?
  *The location respectively the institution where the experiment was conducted, is of interest as regional characteristics might influence the data.*
7. *What* was the order of the stimulation parameters in a particular experiment?
  *The order of the particular approaches influences the results as there might be effects from the timing of the experiments or the duration since their preparation. That means, with respect to the evaluation of the results, we are interested in this order.*

## Related work

The provenance of research data including their research investigations combines several research fields, ranging from general-purpose methods and standards for the documentation of provenance to specifically tailored methods and platforms for the tracking of research and other activities. In the following, we will discuss recent work within those fields and relate it to our method.

Many methods aiming at documenting the provenance of activities have already been proposed. Here, we consider the classification of provenance information following the definition of Herschel et al. [12] and Lim et al. [13]:

1. *prospective* provenance describes “*an abstract workflow specification as a recipe for future data derivation*” [13],
2. *retrospective* provenance documents a “*past workflow execution and data derivation information, i.e., which tasks were performed and how data artifacts were derived.*” [13], and
3. *evolution* provenance illustrates “*the changes made between two versions of the input*” [12]. In other words, versions of the procedure, the data or the parameters are reflected by evolution provenance similar to version control such as implemented by Git for source code.

Applying those definitions to the use case at hand, prospective provenance allows to keep track of changes of laboratory specific operating procedures in general, while retrospective provenance allows to document the actually executed sequence of activities that resulted in a particular set of research data. At last, evolution provenance allows to track changes made to the actual ELN protocol or the inventory database items.

With respect to the research workflows to be represented by provenance modeling, two different types can be distinguished:

1. *In-silico studies* employ computational methods for the analysis of the data. Workflow systems like Taverna [14], Kepler [15], or Galaxy [16], but also programming environments like Jupyter Notebooks [17] have been successfully augmented to record retrospective provenance.
2. *Wet-lab experiments* are courses of activities in a laboratory. While several approaches exist that describe prospective provenance [18, 19] by analysing published protocols, only little work is done on documenting retrospective provenance for these workflows.

More detailed information about provenance modelling and the employed methods are provided in the literature [3, 12]. Here, we are interested in providing detailed information about the origin of research data. Thus, we aim at providing retrospective provenance documentation of research data from ELN protocols documenting wet-lab experiments.

The *Smart Tea* project [20] similarly aims at the semantic metadata recording for research data from within a customized ELN. The developed ELN provides a structured graphical user interface requiring the user to provide information for predefined variables. All information is directly transferred into a linked data representation and persistently archived with a linked data server. While this approach perfectly guides users through the sequence of activities and tracks retrospective provenance at the same time, it fails to keep track of deviations from the predefined plan. Furthermore, as the documentation is directly translated into a semantic representation, additional information that was not considered before can hardly be attached to such protocols, which restricts both the expressivity of the semantic model and the user to previously known information.

Similar to the Smart Tea project, the PROV templating approach [21] suggests the recording of provenance information given a pre-defined provenance model. In other words, the main idea is that applications only store values for placeholders in a particular provenance model which was shown to be more efficient than the storage of the original provenance models [21]. This solution is very efficient if a very large number of identical provenance structures with some variable information are to be stored. If, however, the application requires more flexibility in terms of the provenance structure, the template approach does not utilize this efficiency advantage. Note that provenance templates encode a semantic representation with variables whereas protocol templates provide guidelines for experiments.

Curcin et al. [22] use a very similar approach for the provenance modelling in diagnostic decision support systems. A more flexible approach are Knowledge Graph Cells (KGCs) proposed by Vogt et al. [23]. They provide a concept for the definition of knowledge structures. In particular, rules including ABox and TBox expressions might be defined that allow the dynamic modification of the KG. Thus, KGCs might be used to specify potential semantic structures of ELN protocols without particular information inside. The application of KGCs would require a complete definition over all possible semantic representations of ELN protocols, which is infeasible.

With respect to the vocabulary used to semantically describe the laboratory specific information, the EXperimental ACTions (EXACT2) ontology together with the Natural Language Processing (NLP) framework [18] aims at the automatic extraction of knowledge from biomedical protocols for prospective provenance. Similarly, the SeMAntic RepresenTation for Experimental Protocols (SMART Protocols) ontology reuses EXACT2 to represent prospective provenance from published protocols [19]. In contrast to both approaches that represent a plan, we aim at retrospective provenance, i. e., a particular course of activities. Both approaches, however, could be used to describe prospective provenance of the underlying plan of an ELN protocol, to allow the documentation of potential deviations from the original plan. The Reproduce Microscopy Experiments (REPRODUCE-ME) ontology [24] introduces a specific vocabulary to describe retrospective provenance for microscopy experiments. Besides, the domain-independent ontologies, PROV-O and its predecessor Open Provenance Model (OPM) [25] are frequently employed as upper level ontology for provenance documentation [3]. Furthermore, many extensions for specific applications have been proposed, e. g., the Provenance, Authoring and Versioning (PAV) ontology proposes a mechanism for the versioning and authoring of web resources [26] and CollabPG encodes collaborations within processes [3]. With respect to the application domain of the use case, the Open Biological and Biomedical Ontology (OBO) Foundry is an community initiative aiming at the development and maintenance of ontologies in the biomedical domain [27]. The Basic Formal Ontology (BFO) [28] is the upper level ontology that is used for each of the OBO ontologies.

For the retrospective provenance documentation of research data from computational workflows, several specifically tailored tools and approaches have been proposed in the literature. ProvBook [17], for instance, tracks provenance in Jupyter notebooks that are used for literate programming; Dataprov [29] is a wrapper tool producing provenance information from the execution of analysis tools; and noWorkflow [30] captures provenance information from analysis scripts such as for the programming language Python. Beside these methods, other provenance tracking approaches known as lineage retrieval [31] or lineage tracking and workflow systems exist [32]. In general, in-silico workflow systems not only record provenance information, but at the same time specify the involved processing steps and enable their execution possibly on a distributed system [33]. However, as these systems are limited to tackling computational analyses, their usage for the provenance of research data from wet-lab experiments is difficult.

Regarding the completeness of the documentation with respect to reproducibility, plenty of standards exist that aim at the definition of the minimum set of information required to comprehend and reproduce the research investigation for different applications. With respect to the use case at hand, the minimum information for electrical cell stimulation [34] and the Minimum Information About a Cellular Assay (MIACA)^2^ provide such references for the documentation. Similarly, Standard Operating Procedures (SOPs) or published instructions for experiments encode standards for the documentation of a particular experiment.

When considering the publication or archival of research data, metadata is important to provide additional context, enabling others (including the future self) to understand the research process and the resulting data. In particular, the FAIR guiding principles provide abstract recommendations for handling research data to enable its re-usability [1]. Together with the implementation suggestions of these guidelines [2], they provide a framework which is also applicable for research data from wet-lab experiments. While both guidelines provide generic recommendations regarding research data documentation, different standards exist that provide vocabulary for their support. Several initiatives foster the development of documentation standards for research data including the Data Documentation Initiative (DDI) that focus on standardising metadata for social science datasets [35]. The Dublin Core, instead, is a more general definition of 15 metadata elements for electronic resources [36, 37]. Similarly, Data Catalog Vocabulary (DCAT), provides a common vocabulary for the interoperability of data catalogs [38] and, thus, also defines required metadata for research data. Additionally, domain-specific metadata standards have been developed. With respect to the use case, this includes metadata for microscopy images such as proposed by the RDM4mic Initiative^3^. In addition to these metadata, the information inside the data file might also be described. For this purpose, codebooks and data dictionaries are employed [39, 40]. Considering a CSV file as an example, this includes information about each column such as the domain of the values and the unit of the measurements. This information is defined in a separate file that helps comprehending the raw data.

For the publication and archival of this data including the semantic documentation, several approaches have been proposed. These include bundling formats such as BagIt [41], Oxford Common File Layout (OCFL) [42], and RO-Crate [43], but also literate programming methods such as Jupyter Notebooks (https://jupyter.org/) combine (parts of) research data, their analysis source code and results as well as their documentation. RO-Crate [43] is a mechanism that allows to bundle resources together with their associated metadata supporting the FAIR publication and archival of the research data. By re-using existing vocabulary such as schema.org or PROV-O it implements a linked data approach to enable researchers to provide all information necessary to (re-)use the described research data. This includes basic properties such as author and title of the resource, a license for publication, or a description of the files, but also a description of the workflow used to create those files in terms of retrospective provenance including employed software and other equipment. In brief, a RO-Crate bundle consists of the research data file and a metadata file called ro-crate-metadata.json that contains structured metadata about the files and the entire bundle in a JSON-LD format. While the ro-create-metadata.json contains all information in machine interpretable way, it is accompanied by a human readable HTML representation. RO-Crate has successfully been used for the documentation of retrospective provenance of in-silico studies [44], but can, due to the flexibility of the vocabulary, also be used for retrospective provenance of wet-lab experiments.

## Methods

The objective of the study was to investigate whether it is possible to create semantic documentation of the research process and the resulting research data in terms of provenance. To this end, semantic documentation was manually created by analysing the ELN protocol. To support potential automation of the semantic model creation, based on the results of this analysis, a protocol template was designed that (i) guides researchers through the process while (ii) requiring them to provide all information necessary to comprehend the origin of the research data. The resulting protocol template was split up into a set of templates that encode steps of an experiment such as the staining or the imaging with a particular set of stimulation parameters. These sub-templates ease the re-use for new experiments e. g., by combining them in other permutations. Based on this, researchers documented their wet-lab experiments, resulting in a set of ELN protocols, each of which contains variations, such as differences in parameters, execution time, or execution order. The different protocols were then automatically analysed, translated into a semantic model and finally bundled into self-contained archives. In the following, a detailed description of each step is provided.

### Manual model engineering

The manual engineering process for the semantic model of the ELN protocol was comprised of iterative modelling and reviewing. Domain experts were consulted during this process in order to validate the model. The main objective of this process was to check if all information for the semantic provenance modelling are available in ELN protocols and whether they can be transferred into a semantic representation by employing existing ontologies. The aim of the resulting model was to document the provenance of the research data.

For the model engineering Protegé [45] was used. In particular, the modelling was conducted as follows:

1. BioPortal (https://bioportal.bioontology.org/) and Ontobee (http://www.ontobee.org/) are used to identify relevant ontologies for terms from the ELN protocol and the inventory database items.
2. A set of ontologies is selected from these search results so that the coverage of terms from the ELN in a single ontology is maximised. Ontologies from the OBO Foundry (http://www.obofoundry.org/) [27] compatible with the BFO [28] were preferred.
3. Ontology classes representing inventory database items in the ELN (see Fig. 3) were added into the ELN description of the corresponding inventory database item as a reference for the semantic modelling.
4. The semantic model itself is constructed by ABox statements, i. e., the creation of instances of these classes that represent the particular entities and activities of the protocol and the inventory database. Each instance got a unique identifier in the local namespace reflecting the individual entity, e. g., MG-63_(P25,_LOT_57840088) is used to encode passage 25 of the MG-63 cells that were delivered with the lot number 57840088 (see also Fig. 5). The specific input and output relations of the activity classes were used in order to connect the particular entities correspondingly.
5. References to the same entities in other KGs such as Wikidata [46] were included by employing the owl:sameAs relation. This is essential for linked open data according to the 5-star deployment scheme proposed by Berners-Lee^4^.

The following three rules were considered during the iterative modelling in order to prevent the introduction of a bias from modeller and domain experts: (i) use ontological classes of the same granularity as the terms in the experiment documentation, e. g., “*washing*” instead of “*material processing*”, (ii) avoid the introduction of new classes and attributes whenever possible (avoid TBox statements) and re-use existing ontologies [47], and (iii) use only information from the ELN protocol and do not introduce further knowledge despite the references to other KGs. Thus, the semantic model serves as demonstrator for the inherent potential of ELN protocols.

### Structure-based modelling approach

The manual model engineering reveals the potential of ELN protocols for the semantic documentation of research data. However, in order to use this at large scale, a more automated approach is needed. To approach this target, the structure-based method presented here employs the textual structure in the ELN protocols as well as basic text analysis which is introduced in the following sections.

Considering the ELN protocol from the manual model, we observed that the main content is structured by:

- headings and paragraphs,
- tables (table headings and body),
- enumerations and lists, and
- links to inventory items and research data.

Headings are used to structure the documentation, e. g., the general section about the experimental details or a particular set of activities are preceded from a heading (upper respectively lower part in Fig. 2). In the latter case, different sets of activities in a protocol correspond to the templates we extracted, i. e., at each headline a new template was included.

Tables are used here for two different purposes:

1. *Key-value mappings* are tables that encode general information about an experiment or inventory item, e. g., the objective of the investigation or the manufacturer of a resource. The description of inventory items mainly consists of a table of this kind (see Fig. 3).
2. *Lists of activities* are tables with two columns “*Step*” and “*Starting time*”. Each row encodes an atomic activity of the experiment (see Fig. 2).

Especially for the activity tables, cells include also enumerations, lists and paragraphs further describing the atomic activities and parameters but also linking inventory items and the research data. As an example, see the last row in the activity table in Fig. 2. Note, that we assume each row defining an atomic activity that we do not split up at this stage.

Considering our ultimate goal of retrospective research data provenance documentation, we exploited the structure of the ELN protocol as follows:

1. General information such as the researcher conducting the experiment, but also the objective of the investigation are parsed from the key-value table at the beginning of the protocol. This information is added to the protocol activity using the relation qualifiedAssociation (prov:qualifiedAssociation).
2. Activities described within the ELN protocol are hierarchically structured to represent different levels of granularity. The top level activity resembles the entire experiment, while the different main sections are represented by second level activities. Note that each main section contains an activity table. Finally, the third level represents activities from table rows of those tables.
3. All activities are augmented by inventory items mentioned in the respective description by the used (prov:used) relation.
4. For each research data file created during the investigation, a corresponding entity is created. Assuming that the mention of a file inside an activity marks the creation of this file, the activity is linked to the file by using the wasGeneratedBy (prov:wasGeneratedBy).

As previously described, we do not further split up the third level activities, i. e., complex structures such as enumerations and lists including their order inside a step description are taken as atomic.

Beside the use of structural elements in the ELN, which was the base for the manual model, we identified different repeating patterns that can be exploited.

From the textual description of activities such as “*incubate 5min in [Device] SANYO CO2 Incubator at* 37^∘^*C*”, or “*wash cells with [Washing solution] PBS without Ca/Mg [..]*”, we observed the use of verb phrases indicating the activity of the step: “*incubate*” respectively “*wash*”. Here, we use the head verb of those phrases to assign the corresponding ontological class from a prior mapping. Similarly, information about researchers and institutions, manufacturers and file mime-types as well as experiment type are included. For large scale usage, these information might also be retrieved from an organizational or research information system.

Parameters that are used in the textual description are identified by their unit, e. g., “*1.5ml*”, “*5min*”, and “37^∘^*C*” by employing regular expressions. They are then represented as blank nodes connected to the step using the relation has value specification (OBI_0001938 ) with the value as numerical value of the parameter and the unit connected by has measurement unit label (IAO_0000039 ). We observed that most of the units mentioned in the protocols at hand are defined in the Units Ontology (UO) [48].

Another frequently used pattern observed in the textual description is the mixture of biological and chemical resources, e.g., “*89% [Culture Medium] DMEM + 10% [Serum] FCS + 1% [Antibiotic] Gentamicin*”. By employing the following regular expression, the contained information is extracted and transferred into a representation of activity of type creating a mixture of molecules in solution (OBI_0000685 ): 

Depending on the appearance of attribution notes in the corresponding contexts (e. g., “*(Attributed to Susanne Staehlke)*”), we create separate activities following the same specification. Figure 7 contains an example activity encoding the creation of the above mixture.

### Preparing ELN protocol template

ELN protocols encode instructions (i. e., lists of activities) to (re-)produce the particular research findings. This does not restrict researchers but rather provides a guideline based on earlier experiments. Specifically, they include parameters, timestamps, and the research data. Taking the first experiment of our study which was documented as an ELN protocol, we derived a protocol template by marking all variable information as placeholders. Together with the domain experts, this generalisation has been validated to allow the usage as a basis for new experiments. The main advantage for the researchers conducting experiments in the wet-lab is that all parameters that need to be documented during the experiment are highlighted while the overall description of the process is already done. Thus, errors introduced from missing parameters or instructions are reduced. If, however, the documentation needed to be modified during the experimental execution, researchers can adjust the activities and description.

**Fig. 4:**
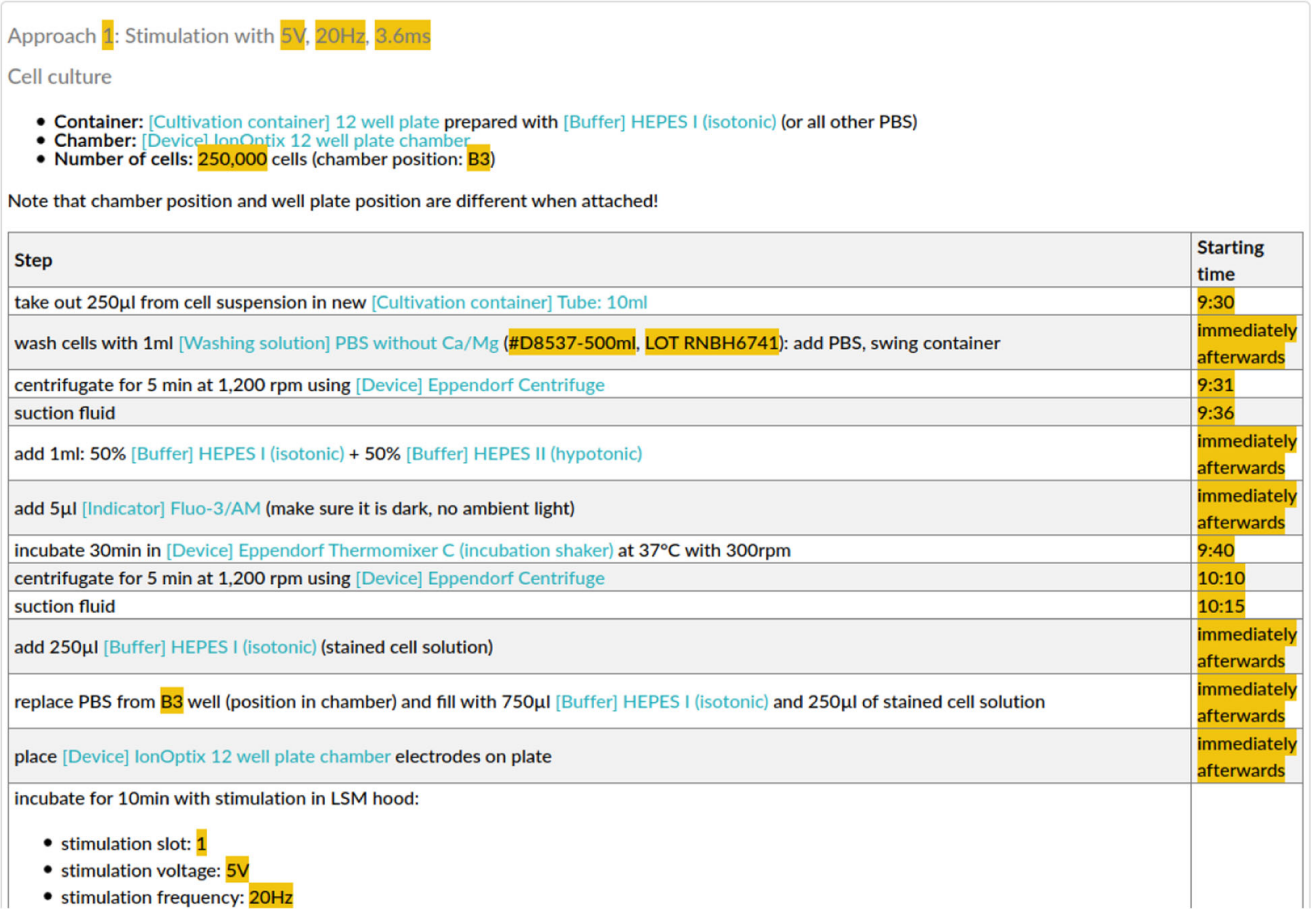
Template transferred from an ELN protocol section by highlighting parameters (marked with orange background color). The template contains the preparation and microscoping of a sample with stimulation. Note that this template aims at supporting researchers during their documentation, but the semantic translation approach is more general

This protocol template might already be used for the documentation of identical experiments (incl. identical ordering of parameter variations). However, as the researchers in our use case permute the different parts of the experiment (i. e., the stimulation parameters in each experiment), the templates were further split up in individual steps. For the use case at hand, we identified the following four parts: (i) Preparation, (ii) Fluo-3 Staining, (iii) Ca-imaging with Stimulation, and (iv) Ca-imaging without Stimulation. Figure 4 illustrates the template for the approach using electrical stimulation. Placeholders that will be replaced with specific parameter values during an experiment are marked with orange background color. These templates can be re-combined and used to encode new experiments. A *protocol template*, therefore, can be interpreted as a combination of templates which themselves are combinations of activities in a textually structured description. In consequence, an ELN protocol represents a completed protocol template with actual parameters.

### Bundling research data and re-use

The structure-based approach automatically translates the ELN protocol into a semantic representation of the activities and resources involved in the production of the research data. In order to combine this semantic representation (i. e., the documentation) with the research data, we employ the RO-Crate format. The RO-Crate bundle consists of the semantic model in a JSON-LD file ro-crate-metadata.json, the research data files as well as a human readable copy of the original ELN protocol and the inventory item description as HTML files.

By using the resulting RO-Crates for our use case, we answer the raised provenance questions. Therefore, we load all semantic representations from the RO-Crates into a linked data server with a SPARQL endpoint. In this study, we use the Apache Jena Fuseki (The Apache Software Foundation, https://jena.apache.org/, v4.1.0) for this purpose.

An advantage of the semantic representation of the research data documentation is its machine-interpretability. This enables to compare the experimental processes with respect to similarities and potential differences that may have influenced the final result. This includes the particular execution times, but also omitted or additional steps as well as different parameter combinations. Furthermore, influences of the order of the different parts can easily be investigated (W7).

## Results

First, we present the details of the manually engineered semantic representation of the Ca-imaging procedure which served as (i) a proof of concept for the effectiveness of retrospective provenance documentation from ELN protocols, (ii) a basis for analysis of the ELN protocol structure, and (iii) the development of the protocol template for research guidance. Second, details of the structure-based semantic translation for the seven Ca-imaging protocols with stimulation are given. Finally, we present the results of the evaluation of the RO-Crate bundles.

### Manually engineered model

The semantic representation of the Ca-imaging procedure is based on the upper level ontology BFO. In addition, PROV-O [25] is used for retrospective provenance documentation of the experimental results. Table 1 lists the most important ontologies used in the model. For the representation, an artefact based modelling approach was selected, where artefacts are central to the model and are used to connect activities via their corresponding input and output relations. In total, the protocol as well as the inventory items are represented in about 80 resources of 46 types connected by almost 20 distinct predicates from 13 vocabularies.

**Table 1:** Ontologies selected for the manually engineered model. Upper rows list general ontologies; the lower rows domain specific ontologies for resources and activities

Name	Source	Details
BFO	[28]	Basic Formal Ontology
PROV-O	[25]	PROV Ontology
BTO	[49]	BRENDA Tissue Ontology
CHEBI	[50]	Chemical Entities of Biological Interest Ontology
CLO	[51]	Cell Line Ontology
OBI	[52]	Ontology for Biomedical Investigations
FOAF	http://xmlns.com/foaf/0.1/	People and their web information

**Fig. 5:**
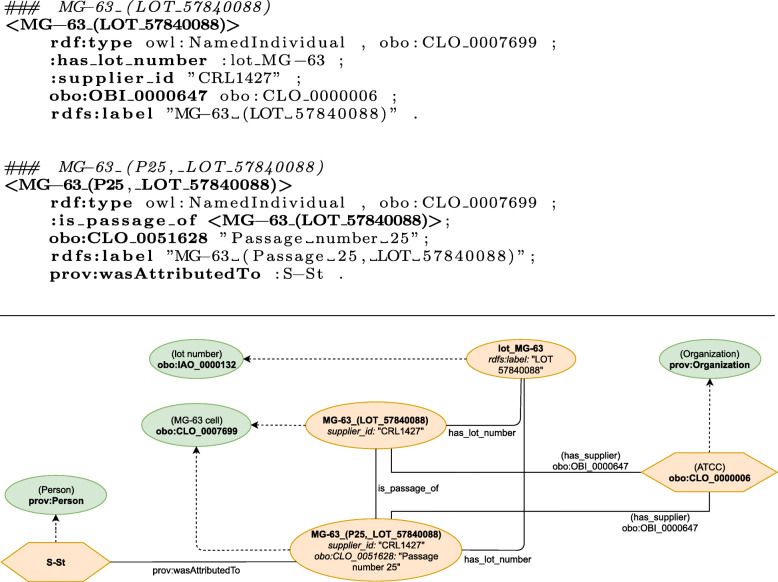
Graphical representation of the manually engineered semantic model of the MG-63 cell line used in the protocol. (Source: Schröder et al. [8])

All inventory items that were mentioned as resources in the protocol were represented by instances of the corresponding ontology classes (ABox statements), which is exemplified in the following by use of the MG-63 cell line. The manually engineered representation as well as the corresponding inventory database description are illustrated in Figs. 5 and 3, respectively.

In the ELN protocol, a passage with number *25* of the originally supplied *MG-63* cells with lot number *57840088* was used: “*[Cell line] MG-63 P25 LOT 57840088*”.^5^ This is modelled by using multiple instances of the corresponding class MG-63 cell (CLO_0007699 ), which are connected with the relation is_passage_of. The passage information are annotated using the attribute passage situation (CLO_0051628 ). lot numbers are represented as an instance of lot number (IAO_0000132 ) and connected to the cell instances using the newly defined relation has_lot_number. The creation of a cell passage is attributed to a researcher using the relation wasAttributedTo (prov:wasAttributedTo). Finally, the supplier is an instance of class Organization (prov:Organization) and related to the cells using has_supplier (OBI_0000647 ).

The modelling of the ELN protocol can be summarized as the creation of instances of activity classes that require their individual input entities and often produce an output entity which serves as an input for the subsequent activity (artefact based modelling). Examples of atomic activities and their corresponding activity classes include washing (OBI_0302888 ), creating a mixture of molecules in solution (OBI_0000685 ), or cell line cell culturing (CLO_0000000 ). The relations that are used to connect the entities to the activities are modelled in the corresponding ontology and depend on the actual activity class. Additionally, these processes are also of type Activity (prov:Activity) in order to encode general provenance information.

**Fig. 6:**
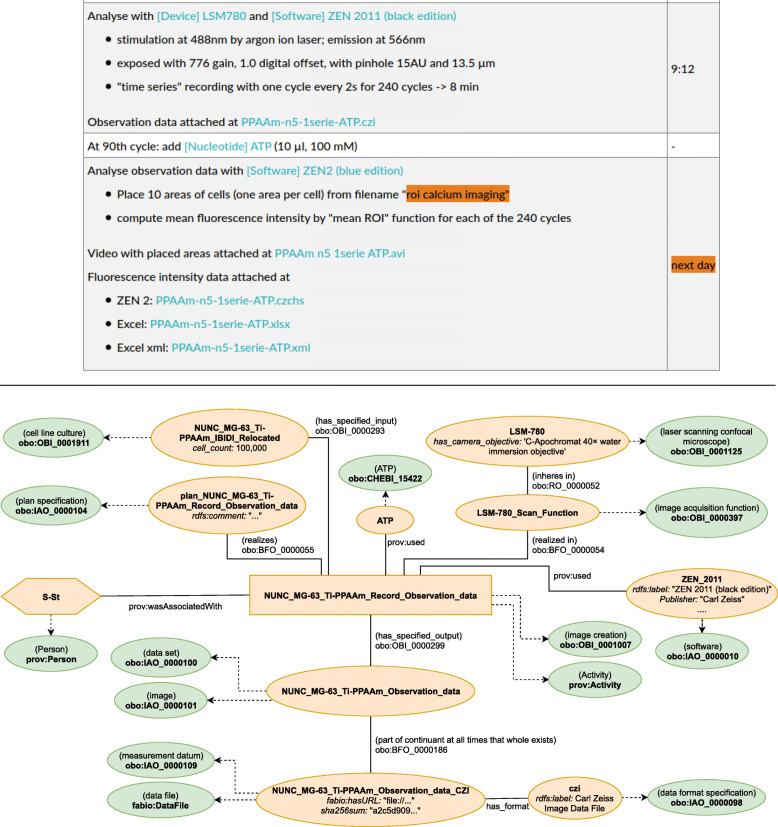
Graphical representation of the semantic model describing the data recording (see also Fig. 5). (Source: Schröder et al. [8])

This modelling approach was employed for the entire ELN protocol. However, the most interesting part when it comes to the provenance documentation of research data is the activity, which produces or uses the research data. The upper part in Fig. 6 illustrates the documentation from the ELN protocol relevant for the research data generation: the first two steps describe the creation of the data while the last step contains the details about the actual analysis.

### Structure-based model

For the structure-based model, an activity based modelling approach was used to resemble the textual structure of the ELN protocol. For this purpose, the model was build upon the general purpose ontologies RO-Crate, PROV-O, and BFO. In total, for the representation of the seven protocols and their corresponding inventory items, 1935 resources of 18 types connected by 36 distinct predicates from seven vocabularies were used.

**Fig. 7:**
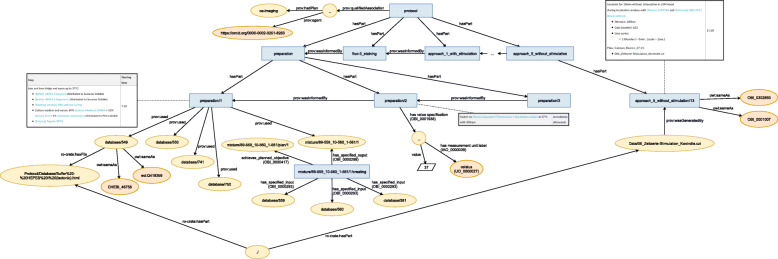
Graphical representation of an excerpt of the semantic model that was created semi-automatically

The structural hierarchy of the activities was represented by bfo:hasPart, while the sequential order was represented by wasInformedBy (prov:wasInformedBy). Figure 7 illustrates this structure. For each activity the general types Action, prov:Activity, and bfo:process were used. Further links to external ontologies were added by owl:sameAs, for instance “*wash*” was augmented by washing (OBI_0302888 ).

The RO-Crate’s root data entity that describes the research data is required to be an entity of type Dataset (schema:Dataset). Thus, research data files are added to this dataset by hasPart (schema:hasPart). The connection of these file entities and the hierarchical structure of the activities is represented by wasGeneratedBy (prov:wasGeneratedBy) (see the right part of Fig. 7), when mentioned in the activities’ textual description. This means that all files are included in this root data entity (via hasPart), but are not necessarily associated to the activities, if they are not mentioned.

Following the RO-Crate specification, ELN inventory database items are encoded as the domain-independent type IndividualProduct as they provide contextual information. However, the ontological knowledge about the type of the biological and chemical resource was added using the relation owl:sameAs by the external references from the description in the ELN. The resulting entity is connected to the activities using used (prov:used). Resources with a specific passage or lot number are added as individual entities connected to a general entity encoding the inventory database item using the relation is_instance_of. Furthermore, attributes has_passage_number and has_lot_number are added with their corresponding information.

Several mixtures are used in the ELN protocols. This information is modelled around the activity creating a mixture of molecules in solution (OBI_0000685 ). All resources that are used in this activity are linked by has_specified_input (OBI_0000293 ) and the resulting mixture entity by has_specified_output (OBI_0000299 ). To specify the recipe of this mixture, a material combination objective (OBI_0000686 ) is created and linked to the activity using achieves_planned_objective (OBI_0000417 ). If an attribution of this mixture is annotated in the ELN protocol, the corresponding agent is associated with the resulting mixture entity via wasAttributedTo. Note that recipes of a mixture are independent of the actual creation activity, i. e., if multiple researchers create a mixture using the same recipe, the same recipe entity is referenced, but individual activities and mixture entities are created.

With respect to parameters, we extracted values and units for the following types: (i) time and duration (*min* and *ms*), (ii) temperature (^∘^*Celsius*), (iii) frequency (*Hz*), and (iv) voltage (*V*) and represented by their corresponding classes. Specifically, the frequency and the voltage are of interested as they provide the parameters for the stimulation of the cells during the Ca-imaging approach.

### ELN protocols and protocol templates

By providing templates for the individual parts of the experiment (preparation, Fluo-3 staining, Ca-imaging with and without stimulation), the researchers were able to compile seven ELN protocols with different permutations of the experiment parameters. In comparison to the predefined protocol template, we observed that the researchers further modified the ELN protocol description to reflect the particular course of activities and observations conducted in the wet-lab, e. g., the repetition of an experimental setting due to issues in the previous experiment or the documentation of issues during the experiment. That means, the model represents such deviations from the original plan (prospective provenance), and allows to track the actually documented activity sequence by means of retrospective provenance.

### Research data bundles

In summary, seven RO-Crates have been created, one for each ELN protocol of the Ca-imaging experiments with stimulation. The corresponding semantic representation was automatically created using the structure-based approach. All research data that was produced in a particular experiment together with this semantic representation was bundled in the RO-Crate. In order to foster readability, a copy of the ELN protocol and the inventory items description was included in the form of HTML files. Thus, the RO-Crates contain between 110 and 135 files and are between 107 and 185 MB large. The particular ELN protocols are encoded in models of 2,174 to 2,553 triples with 15,823 triples in total. As some triples, such as researchers, institutions, and resources are identical across all RO-Crates, the number of unique triples is only 13,490. The number of triples per protocol differ due to deviations in the documentation from the original plan and the number of research files.

**Fig. 8:**
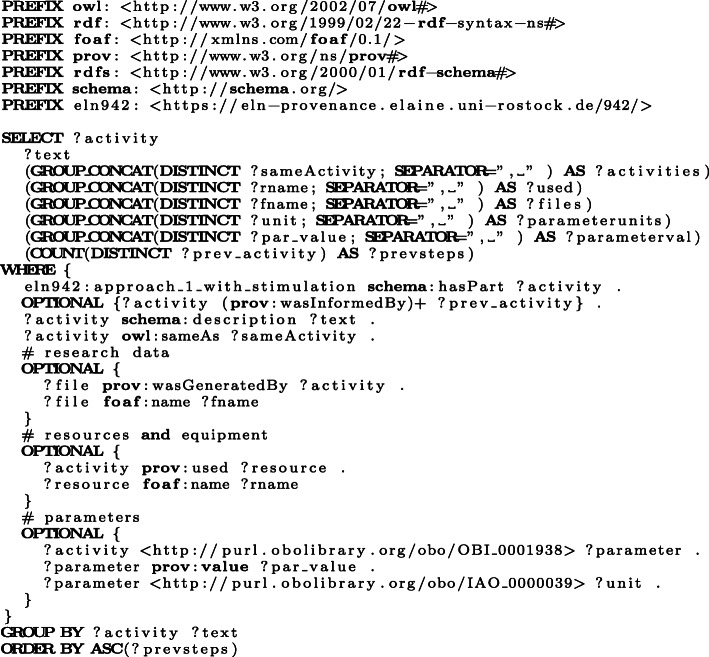
This SPARQL query selects (1) the ontological activity classes, (2) the research data produced, (3) the resources and equipment that is used, and (4) the parameters for each atomic activity order by their execution in a Ca-imaging approach with stimulation from one of the use case ELN protocols that have been translated using the structure-based modelling approach

The structure-based approach employs RO-Crate, PROV-O, and BFO as upper level ontologies. Especially RO-Crate and PROV-O are designed to encode provenance information about resources. Provenance information about experimenter, manufacturer, biological and chemical resources, activities, and research data are transferred by this approach into a semantic representation. To illustrate the capabilities of the resulting RO-Crate bundles, we evaluated SPARQL queries for the W7-questions in our use case (see the Use case section). Considering the question “*How was a particular file created?*” (W3), Fig. 8 presents the corresponding SPARQL query for a Ca-imaging approach in a particular experiment. Table 2 illustrates an excerpt of the result of this query, i. e., the sequence of activities from one experiment, providing the result to the question W3. That is, for every atomic activity within the Ca-imaging approach, the description as well as the created research data are listed in the order of the execution. Moreover, all resources and equipment (W2) as well as the parameters are depicted as result of the query.

**Table 2:** An excerpt of the resulting output for the SPARQL query in Fig. 8

Activity	Text	Act.-Class	Resources	Files	Par.-Units	Par.-Values
[...]						
ap_1_with_stimulation/14	place [Device] IonOptix 12 well plate chamber electrodes on plate	obo:NCIT_C52253	IonOptix 12 well plate chamber			
ap_1_with_stimulation/15	incubate for 10min with stimulation in LSM hood: [...]	obo:OMIT_0005807, obo:OBI_0001007, obo:OBI_0302893	LSM780, ZEN 2011 (black edition)	Data/02_Zeitserie-Stimulation_5V_7.9Hz.czi	obo:UO_0000031, obo:UO_0000028, obo:UO_0000218, obo:UO_0000106	5, 10, 7.9
[...]						

**Fig. 9:**
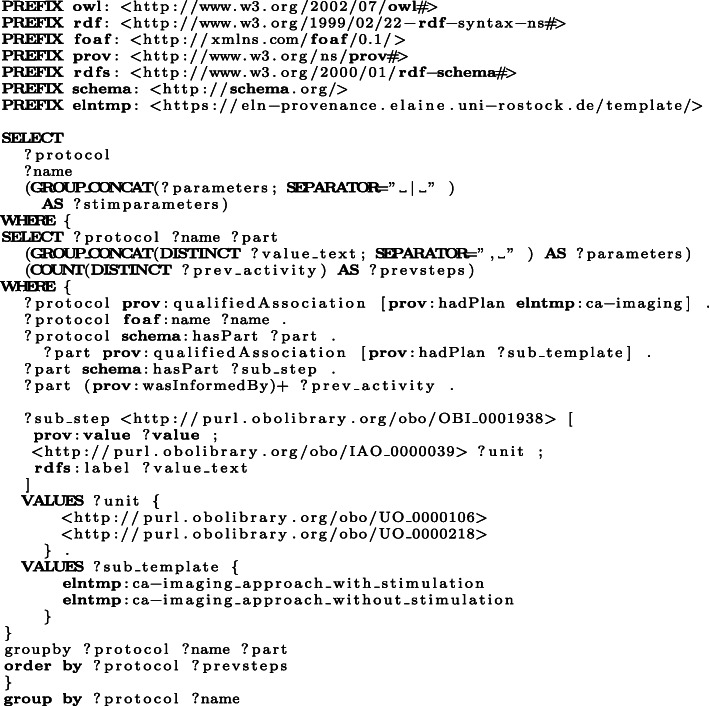
This SPARQL query selects all experiments following the Ca-imaging procedure and collects their stimulation parameters in the order that they have been investigated

Beside queries for individual experiments, the semantic models enable to compare the documentation of multiple experiments. As an example, we consider the question “*What was the order of the stimulation parameters in a particular experiment?*” (W7) that should be answered for seven experiments. Figure 9 illustrates the query for the comparison of multiple experiments based on the order of their stimulation parameters. The corresponding results are shown in Table 3.

**Table 3:** The result for the SPARQL query in Fig. 9 illustrating a comparison of multiple experiments based on the order of their stimulation parameters

Protocol	Title	Stimulation Parameters
eln1124/protocol	Ca-imaging (with stimulation) 29.01.2021	7.9Hz, 1V | 7.9Hz, 5V | 20Hz, 5V | 20Hz, 1V
eln1042/protocol	Ca-imaging (with stimulation)	20Hz, 1V | 7.9Hz, 1V | 7.9Hz, 5V | 20Hz, 5V
eln1021/protocol	Ca-imaging (with stimulation)	20Hz, 1V | 20Hz, 5V | 7.9Hz, 5V | 7.9Hz, 1V
eln1022/protocol	Ca-imaging (with stimulation)	7.9Hz, 5V | 7.9Hz, 1V | 20Hz, 1V | 20Hz, 5V | 7.9Hz, 5V
eln1023/protocol	Ca-imaging (with stimulation) Failed (durch ATP Zugabe hat sich der Bildausschnitt verändert)	7.9Hz, 1V | 7.9Hz, 5V | 20Hz, 5V | 20Hz, 1V
eln942/protocol	Ca-imaging (with stimulation)	7.9Hz, 5V | 7.9Hz, 1V | 20Hz, 1V | 20Hz, 5V
eln1071/protocol	Ca-imaging (with stimulation) 22.01.2021	7.9Hz, 5V | 20Hz, 5V | 20Hz, 1V | 7.9Hz, 1V

The remaining W7-questions could also be validated based on similar queries, as shown in the appendix of this paper. Thus, the proposed approach demonstrates the feasibility of research data documentation using ELN protocols.

## Discussion

The results of the manual modelling show that it is feasible to translate the information of an ELN protocol into a semantic representation for documentation of retrospective provenance of research data. Moreover, it has been shown that the creation of ready-to-publish bundles containing the research data, the associated metadata and the retrospective provenance documentation by using of RO-Crate enables to answer questions about the experimental procedure raising the research data. The manually engineered model implements an artifact based modelling approach that uses ontological terms in full extent. Thus, the resulting representation mainly consists of a sequential list of activities and entities connected via their specific input and output relations. The level of granularity of the model corresponds in most cases to the terms used in the documentation, although, existing ontologies not always provide the same level of detail for all terms. As an example, the terms “relocate”, “transfer”, and “take out” can be subsumed under moving of materials, but still have some distinct differences. Furthermore, when “take out” is used in the context of a fridge or freezer, an ontological modelling additionally requires encoding of the warming up of the material. Thus, providing ontological definitions for these different situations requires much work in future ontological engineering.

In contrast to the manual model, the structure-based approach implements an activity-based modelling mechanism and does not use the specific input and output relations of an activity, but the same activities. As a result, the structure-based approach does not specify the particular role of the used entities. Furthermore, in the manually engineered model, the semantic representation of an entity that results from the sequential execution of activities is difficult without introducing TBox statements. The reason is that this entity needs to reflect the result of the particular activity sequence. In the structure-based approach, these entities need not be defined as the main part of the model consists of a hierarchy of activities including the used resources. This allows the model to represent only the information that is actually contained in the textual description of the ELN protocol, without artificially introducing entities with properties that are the direct result of the activities.

Beside the process documentation, the structure-based approach adds metadata about the mime type, the file size and the checksum allowing to validate the integrity of the research data. This representation of the research data might be extended by additional metadata, which, however, would require the application of file type specific extraction methods, as for instance for the CZI files, or the researchers themselves to provide the information, for instance in the form of data dictionaries for tabular data. Moreover, representing the research data itself in the same representation format as the metadata and the retrospective provenance documentation would enable further data integration and thus allow for automatic data analysis approaches.

Employing the structure-based approach at large-scale, requires knowledge about the relation of terms from the textual description in the ELN to classes and attributes from ontologies. Here, we implemented this relation by a hard-coded mapping, for instance from verb phrases to ontology classes in the case of activities. This can also be achieved by use of a suggestion system for the researchers that proposes ontological classes selected from automated queries of ontological databases. Similarly, external identifier might be augmented. The structure-based approach currently integrates the Open Researcher and Contributor ID (ORCID) and Research Organization Registry (ROR) for persons respectively organizations, but also references to Wikidata entities are used. Several initiatives proposed the use of persistent identifiers for other aspects of wet-lab experiments, e. g., RRIDs (https://scicrunch.org/resources) can be used to reference scientific resources similar to the inventory database of the ELN. While using persistent identifiers, we observed two aspects that are crucial:

1. The granularity of the entity referenced by the identifier needs to be on the same level as is needed for the application. As an example, the organization referenced by the ROR https://ror.org/04dm1cm79does not reflect the particular department that the researchers are affiliated.
2. The entity referenced by the identifier needs to reflect evolution, too. Although the identifier should reference a particular version of an entity, the entity behind might change and, thus, the registry needs to encode these versions and provide corresponding identifier for each version. To the best of our knowledge, this is currently not supported by e. g., RRIDs.

A fine-grained solution for referencing researchers, organizations, and research projects on an institutional level might be implemented by organizational information systems.

Another important aspect is related to privacy protection, for instance, the names of all involved persons in an experiment. While for archival purposes the identity of all involved persons are of interest, it might not be wanted to publish all personal details with respect to privacy protection. The structured representation of the RO-Crate allows to query for all involved persons (W1) and thus would directly allow to easily implement the pseudonymisation via graph update operations.

With respect to the recent advances in information extraction, we employed basic methods. While this does not extract all information of interest, it sketches the potential benefits of automatic text analysis. By employing more sophisticated information extraction methods, for instance, trained on labelled published protocols [53], this could further be improved. This is also true for the extraction of parameters and their assignment to activities as can be seen by recently established NLP challenges such as MeasEval^6^. Moreover, disambiguating detected terms with respect to their context and linking them to the corresponding ontology classes is one of the core challenges in modern NLP.

With respect to the completeness of the documentation of wet-lab experiments, minimal information guidelines provide a reference that can potentially be exploited to create protocol templates. In combination with the proposed structure-based approach, this would allow to employ minimum information checklists using the MINIM model (http://purl.org/minim/description) to enable the validation of the generated documentation.

During the use of ELNs for a longer period of time, inventory database items are regularly updated, because e. g., the supplier changes as well as the software or the firmware of a device is updated. Evolution provenance methods can be employed to represent such changes. In order to reflect these versions also for the research data in the RO-Crate, a data storage solution with versioning is needed. Intra-consortia sharing platforms [54] can be employed for this purpose.

Overall, we have shown that our approach is able to help generate increasingly FAIR data. The ELN Protocols captured together with the data entries in the RO-Crate format increase the findability of data produced in wet-lab experiments creating a binding between experiment steps and data. Likewise, the approach increases accessibility by allowing rich SPARQL queries to be formulated that combine the experiment metadata with the data itself. In terms of interoperability and reusability, the use of common ontologies allows for different experiment runs to be easily compared and documentation to be more easily generated. However, as noted by [55], making FAIR data is not an absolute but a spectrum where there are trade-offs in terms of ability to find and reuse and the effort in documentation. Our approach illustrates this by highlighting the differences between automated capture and manual capture. In particular, while automated capture reduces the burden in capturing FAIR data, it also means, for the time being, the decrease in the richness of the associated metadata needed for reusability. Having a target in terms of manual capture provides a valuable target for automated capture of metadata for the data produced in the wet-lab.

## Conclusion

The presented study investigated the feasibility of creating semantic provenance documentation for research data using ELN protocols from wet-lab experiments. ELN protocols contain specific information about an experiment such as the produced research data but also timestamps, lot and passage number as well as parameters. This is in contrast to templates that serve as general guidelines without such information.

The manually engineered model was used as a proof of concept for the translation of ELN protocols using an Ca-imaging experiment. In order to support researchers in the wet-lab we derived four templates encoding parts of this initial protocol that can be used to create new experiment documentation. Based on these results, a structure-based approach was implemented to translate these protocols into a semantic representation. This approach uses the structure in the description such as headings, tables, and links as well as some basic text analysis. Furthermore, the resulting semantic model is bundled together with the research data. Potential provenance questions from the viewpoint of other researchers using these bundles have been implemented as SPARQL queries in order to evaluate the proposed methodology. We have shown that the structure-based approach in combination with RO-Crate bundling can be used to successfully document research data based on the description in the form of ELN protocols. Thus, these RO-Crates enable the sharing, publication, and archival of the research data in terms of the FAIR principles [1, 2]. Furthermore, in order to guide researchers during the conduction of Ca-imaging experiments, the four derived sub-templates can be combined to provide a documentation basis for new experiments.

Integrating the proposed approach as well as the sketched extensions into a comprehensive Virtual Research Environment (VRE), would enable the tracking of the entire research process and the research data from the creation of a hypothesis to the publication of the data. In particular, the ELN can be used for the documentation of the wet-lab investigation of a research project. The funding information of the research projects including involved researchers and the consortia can be stored in a research information system. Furthermore, the semantic representation of the protocol can be automatically synced with a linked data server as well as the research data be stored in an institutional repository. The particular platforms can be connected with a semantic search interface for researchers that enables searching for similar experiments, data, but also the creation of reports about experimental activities.

## Appendix

### Queries and answers for the w7-questions

Note that for better readability we shortened URIs in some of the following results, e. g., https://eln-provenance.elaine.uni-rostock.de/942/approach_1_with_stimulation/1 has been shortened to ap_1_with_stimulation/1 or http://localhost:3030/Data/02_Zeitserie-Stimulation_5V_7.9Hz.czi has been shortened to 02_Zeitserie-Stimulation_5V_7.9Hz.czi.

#### W1: who participated in the study?


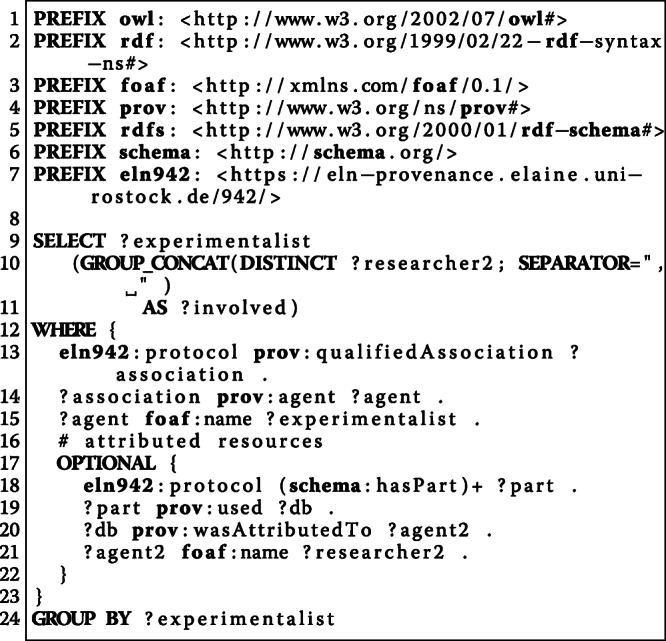

**Table:** 

**Experimentalist**	**Involved Persons**
Susanne Staehlke	Person1 Anonymous, Person2 Anonymous

Note that before publication, we pseudonymised some researchers with respect to privacy protection. Refer to the *Discussion* section for more details.

#### W2: which biological and chemical resources and which equipment was used in the study?


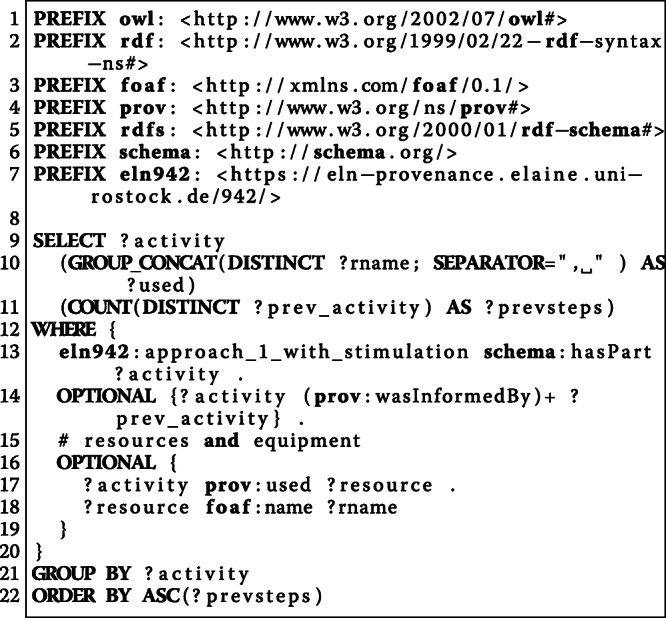

**Table:** 

**Activity**	**Used Resources**	**Number of prev. steps**
ap_1_with_stimulation/1	Tube: 10ml	0
ap_1_with_stimulation/2	PBS without Ca/Mg	1
ap_1_with_stimulation/3	Eppendorf Centrifuge	2
ap_1_with_stimulation/4		3
ap_1_with_stimulation/5	50% HEPES I (isotonic) + 50% HEPES II (hypotonic)	4
ap_1_with_stimulation/6	Fluo-3/AM	5
ap_1_with_stimulation/7	Eppendorf Thermomixer C (incubation shaker)	6
ap_1_with_stimulation/8	LSM780, IonOptix 12 well plate chamber, IonOptix C-Pace EM	7
ap_1_with_stimulation/9	Eppendorf Centrifuge	8
ap_1_with_stimulation/10		9
ap_1_with_stimulation/11	HEPES I (isotonic)	10
ap_1_with_stimulation/12	12 well plate, PBS without Ca/Mg	11
ap_1_with_stimulation/13	HEPES I (isotonic)	12
ap_1_with_stimulation/14	IonOptix 12 well plate chamber	13
ap_1_with_stimulation/15	LSM780, ZEN 2011 (black edition)	14
ap_1_with_stimulation/16	IonOptix 12 well plate chamber	15
ap_1_with_stimulation/17	LSM780,ATP, ZEN 2011 (black edition)	16

#### W3: how was a particular file created?


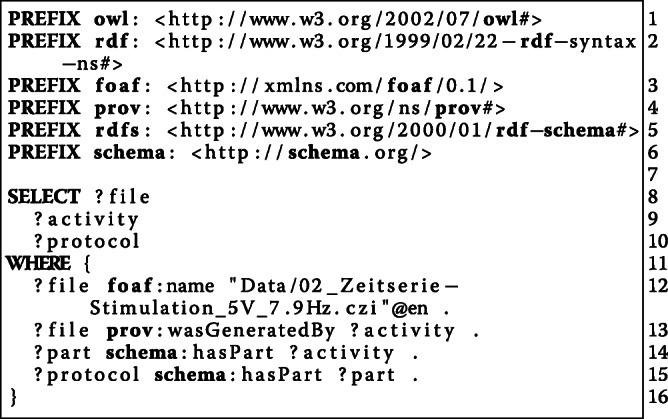

**Table:** 

**File**	**Activity**	**Protocol**
Data/02_Zeitserie-Stimulation_5V_7.9Hz.czi	eln942:ap_1_with_stimulation/15	eln942:protocol

#### W4: when was an activity conducted?


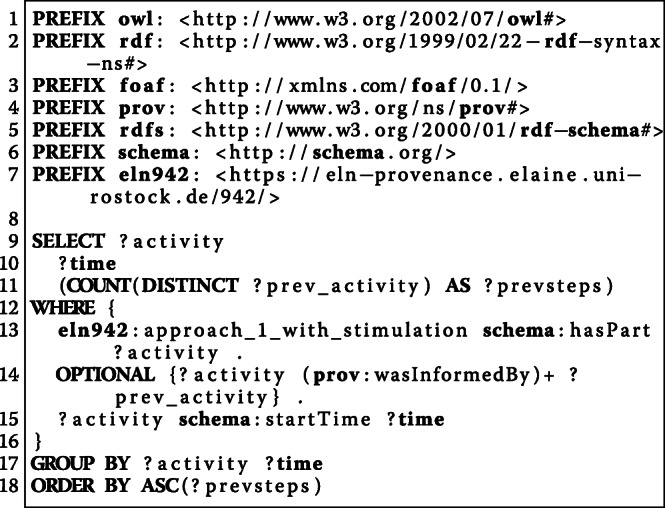

**Table:** 

**Activity**	**Starting time**	**Number of prev. steps**
ap_1_with_stimulation/1	09:00:00	0
ap_1_with_stimulation/2	immediately afterwards	1
ap_1_with_stimulation/3	09:01:00	2
ap_1_with_stimulation/4	09:06:00	3
ap_1_with_stimulation/5	immediately afterwards	4
ap_1_with_stimulation/6	immediately afterwards	5
ap_1_with_stimulation/7	09:10:00	6
ap_1_with_stimulation/8	immediately afterwards	7
ap_1_with_stimulation/9	09:40:00	8
ap_1_with_stimulation/10	09:45:00	9
ap_1_with_stimulation/11		10
ap_1_with_stimulation/12	immediately afterwards	11
ap_1_with_stimulation/13	immediately afterwards	12
ap_1_with_stimulation/14	immediately afterwards	13
ap_1_with_stimulation/15	09:50:00	14
ap_1_with_stimulation/16	10:00:00	15
ap_1_with_stimulation/17	immediately afterwards	16

#### W5: why was the experiment done?


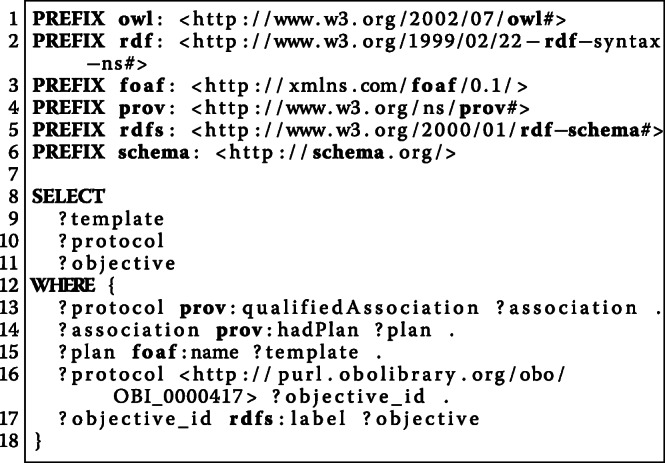

**Table:** 

**Template**	**Protocol**	**Objective**
Ca-imaging	eln1023/protocol	Intracellular calcium dynamic caused by electric fields
Ca-imaging	eln1021/protocol	Intracellular calcium dynamic caused by electric fields
Ca-imaging	eln942/protocol	Intracellular calcium dynamic caused by electric fields
Ca-imaging	eln1071/protocol	Intracellular calcium dynamic caused by electric fields
Ca-imaging	eln1042/protocol	Intracellular calcium dynamic caused by electric fields
Ca-imaging	eln1124/protocol	Intracellular calcium dynamic caused by electric fields
Ca-imaging	eln1022/protocol	Intracellular calcium dynamic caused by electric fields

#### W6: where was the experiment conducted?


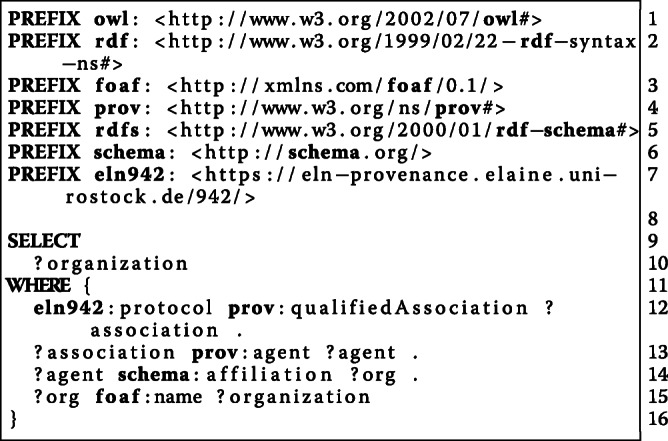

**Table:** 

**Organization**	
University Medical Center Rostock	

Note that the granularity of external identifiers is not always sufficient, e. g., the researchers and the equipment are affiliated in the “*Department of Cell Biology*” which is part of the “*University Medical Center Rostock*”. See the discussion on this issue in the *Discussion* section.

## Data Availability

The manually engineered model is published at https://github.com/SFB-ELAINE/Semantic-Modelling-CA-Imaging. The results of the structure-based modelling approach and the research data are published as RO-Crates bundles at https://github.com/SFB-ELAINE/Ca-imaging-RO-Crate. The source that builds the model based on the ELN API is available at https://github.com/m6121/Structure-based-ELN2LOD. This study is based on the previously published data by Staehlke and Nebe [56].

## Abbreviations

BFO: Basic Formal Ontology
DCAT: Data Catalog Vocabulary
DDI: Data Documentation Initiative
ELN: Electronic Laboratory Notebook
EXACT2: EXperimental ACTions
FAIR: Findable, Accessible, Interoperable, and Reuseable
KG: Knowledge Graph
KGC: Knowledge Graph Cell
MIACA: Minimum Information About a Cellular Assay
NLP: Natural Language Processing
OBO: Open Biological and Biomedical Ontology
OCFL: Oxford Common File Layout
OPM: Open Provenance Model
ORCID: Open Researcher and Contributor ID
PAV: Provenance, Authoring and Versioning
PROV-O: PROV Ontology
REPRODUCE-ME: Reproduce Microscopy Experiments
RO-Crate: Research Object Crate
ROR: Research Organization Registry
RRID: Research Resource Identifiers
SMART Protocols: SeMAntic RepresenTation for Experimental Protocols
SOP: Standard Operating Procedure
UO: Units Ontology
VRE: Virtual Research Environment

## Footnotes

1 A lot number is an identifier for a particular set of materials produced by one manufacturer. Thus, lot numbers enable to track information about the provenance of these material productions.

2 http://miaca.sourceforge.net/

3 https://github.com/German-BioImaging/RDM4mic/tree/master/metadata/templates

4 see https://dvcs.w3.org/hg/gld/raw-file/default/glossary/index.html

5 Note: this is not part of the inventory item description, as this aims at the general cell specification. However, the particular information for a specific experiment are part of the ELN protocol.

6 https://competitions.codalab.org/competitions/25770
